# HIGH IMPACT ACTIVITIES: ASSESSING THE ASSOCIATION BETWEEN SOCIAL PARTICIPATION FREQUENCY AND COGNITION

**DOI:** 10.1093/geroni/igae098.2095

**Published:** 2024-12-31

**Authors:** Eric Vogelsang, Sara Moorman

**Affiliations:** California State University San Bernardino, San Bernardino, California, United States; Boston College, Chestnut Hill, Massachusetts, United States

## Abstract

There is growing recognition that social participation may help attenuate cognitive decline in older ages. Unfortunately, previous empirical research often relies on social participation “summary indexes”, which may obfuscate associations between cognition and specific social activities. Moreover, there has been little consideration as to whether increased social participation frequency is associated with greater cognitive functioning. We utilize two nationally representative longitudinal surveys of older adults: the Health and Retirement Study (21,705 observations across six waves) and the National Social Life, Health & Aging Project (5,317 observations across two waves). Regression models test how associations between social participation frequency and three cognition measures (a) differ by summary indexes; and (b) differ among specific social activities. We also compare and contrast the ways in which social participation is evaluated in these studies. When using summary participation indexes, for both datasets, each additional social activity and each increase in activity frequency was associated with greater cognitive functioning. However, when segregating social activities, we found disparate associations with cognition. Specifically, volunteering (in one dataset) and social/club membership (in the other) were the only activities whose frequency was positively associated with each cognition measure. Committing to regular social and community activity in older ages may help mitigate the risk of cognitive decline. However, caution is advised when interpreting the health effects of social activity counts or aggregate frequency measures. Surveys would benefit by establishing consistent social participation measurement standards.

